# Seroepidemiology of Hepatitis E Virus Infection in General Population in Rural Durango, Mexico

**DOI:** 10.5812/hepatmon.16876

**Published:** 2014-06-01

**Authors:** Cosme Alvarado-Esquivel, Luis Francisco Sanchez-Anguiano, Jesus Hernandez-Tinoco

**Affiliations:** 1 Biomedical Research Laboratory, Faculty of Medicine and Nutrition, Juarez University of Durango State, Durango, Mexico; 2 Institute for Scientific Research “Dr. Roberto Rivera Damm”, Juarez University of Durango State, Durango, Mexico

**Keywords:** Hepatitis E Virus, Seroepidemiologic Studies, Rural Population, Risk Factors, Mexico

## Abstract

**Background:**

The seroepidemiology of hepatitis E virus (HEV) infection in rural areas in Mexico has been poorly studied.

**Objectives:**

The aim of the study was to determine the seroprevalence and correlates of anti-HEV IgG antibodies in adults in rural areas in Durango, Mexico.

**Materials and Methods:**

We performed a cross-sectional study to determine the frequency of anti-HEV IgG antibodies in 273 adults living in rural Durango, Mexico using an enzyme-linked immunoassay. In addition, we searched for an association of HEV exposure with the socio-demographic and behavioral characteristics of the subjects studied.

**Results:**

One hundred (36.6%) of the 273 rural adults (mean age: 39.85 ± 17.15 years) had anti-HEV IgG antibodies. Multivariate analysis of socio-demographic and behavioral characteristics of the participants showed that HEV exposure was associated with increasing age (OR = 1.04; 95% CI: 1.04-1.05; P < 0.001), consumption of untreated water (OR = 1.92; 95% CI: 1.06-3.46; P = 0.03), and availability of water at home (OR = 1.87; 95% CI: 1.07-3.27; P = 0.02). In contrast, other socio-demographic and behavioral characteristics including educational level, occupation, socio-economic status, foreign travel, consumption of unwashed raw fruits, consumption of raw or undercooked meat and raising animals did not show associations with HEV exposure.

**Conclusions:**

The seroprevalence of HEV infection found in rural Durango is higher than those reported in other Mexican populations. Consumption of untreated water is an important factor for HEV exposure in rural areas in Durango. The correlates of HEV seropositivity found in the present study can be used for an optimal planning of preventive measures against HEV infection.

## 1. Background

Infections with hepatitis E virus (HEV) are present worldwide (1-4). HEV is the most common cause of acute viral hepatitis in the world (5). The major route of HEV infection is the fecal-oral transmission (3, 6). Outbreaks of acute hepatitis E have occurred through contaminated water (7). Infections with HEV may also occur by blood transfusion (8). Most infections with HEV are clinically unapparent (8), and acute hepatitis E is usually a self-limited disease (9). However, infections with HEV may lead to severe disease including fulminant hepatitis in pregnant women (10). Chronic hepatitis E cases have been reported in immunosuppressed patients (11). In addition, cases of HEV-related cirrhosis have been described (6, 12). Cirrhotic patients suffering from infection with HEV experience high mortality rates (13).

Very little is known on the seroepidemiology of HEV infection in rural adults in Mexico. Suboptimal sanitary conditions are common in rural Mexico, and such unfavorable conditions may facilitate transmission of HEV among the rural population. Many rural communities in Mexico have poor availability of drinkable water, poor disposal of excretes, soil flooring and overcrowding at home. In addition, infections with HEV are unrecognized because there is a lack of laboratory assays for diagnosis in rural practice in Mexico. 

## 2. Objectives

We sought to determine the seroprevalence of anti-HEV IgG antibodies in adults in rural Durango, Mexico. Furthermore, socio-demographic and behavioral characteristics of the rural subjects associated with HEV seropositivity were investigated.

## 3. Materials and Methods

### 3.1. Study Design and Study Population

**Figure 1.**
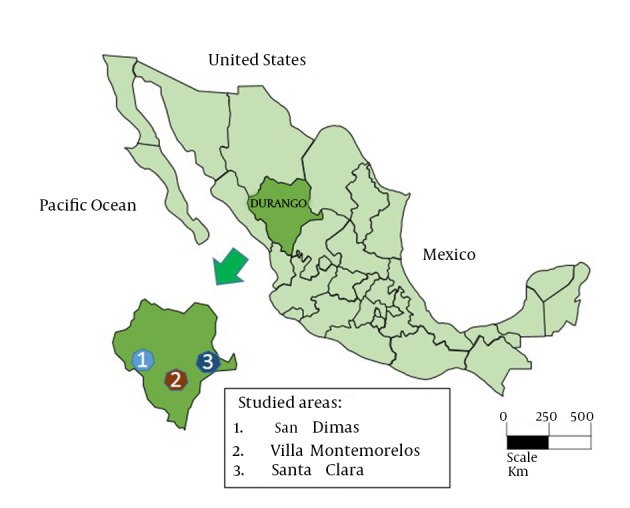
Geographical Locations of the 3 Studied Rural Areas in Durango State, Mexico

We performed a cross-sectional study using stored serum samples from a *Toxoplasma gondii* survey (14). Serum samples were originally used to determine the seroepidemiology of *Toxoplasma gondii* in rural populations in Durango, Mexico and were collected from December 2006 to August 2007. Three rural communities were studied: San Dimas, Villa Montemorelos, and Santa Clara (Figure 1). Inclusion criteria for the study subjects were:

1. inhabitants of rural Durango;
2. aged 18 years and older;
3. any gender; and
4. who accepted to participate in the survey.

Exclusion criteria were: participants with insufficient amount of serum and incomplete socio-demographic data. In total, 273 subjects were included in the study, 152 were inhabitants of San Dimas; 111 were inhabitants of Villa Montemorelos, and 10 were inhabitants of Santa Clara.

### 3.2. General Epidemiological Characteristics of Rural Adults

We obtained the socio-demographic and behavioral characteristics of the participants with the aid of a standardized questionnaire. Socio-demographic data included age, birth place, residence, educational level, socio-economic status, employment, and housing conditions. We used the Bronfman’s criteria (15) to determine the housing conditions and this tool allowed us to assess crowding and sanitation. Briefly, five variables were evaluated: number of people living at home, number of rooms in the house, floor material used in the house (ceramic, concrete, soil), availability of drinkable water (within the house, out of the house), and form of elimination of excretes (flush toilet, latrine). Besides, educational level (years of education) of the head of the family was obtained. Behavioral data included animal contacts, foreign travel, contact with soil (gardening or agriculture), consumption of unpasteurized milk or untreated water, consumption of unwashed raw vegetables or fruits, frequency of eating away from home (in restaurants or fast food outlets), consumption of raw or undercooked meat, type of meat consumed (pork, lamb, beef, goat, boar, chicken, turkey, rabbit, venison, squirrel, horse or other) and consumption of dried or processed meat (chorizo, ham, sausages or salami). In addition, we obtained clinical data including history of blood transfusions or transplants.

### 3.3. Laboratory Tests

We analyzed the serum samples of the participants for anti-HEV IgG antibodies by a commercially available enzyme immunoassay “HEV-IgG ELISA” kit (Diagnostic Automation Inc., Calabasas, CA). The assays were performed following the instructions of the manufacturer. Positive and negative controls were included in each assay. According to the information included in the kit’s insert, the immunoassay used has a sensitivity of 99.8% and a specificity of 99.8%. 

### 3.4. Statistical Analysis

The statistical analysis was performed with the aid of the software Epi Info version 3.5.4 and SPSS version 15.0. For calculation of the sample size, a reference seroprevalence of 10.5% (16) as the expected frequency for the factor under study, 250000 as the population size from which the sample was selected, an absolute error of 4.0%, and a 95% confidence level were considered. The result of the sample size calculation was 225 subjects. The strategy for sampling was firstly providing information about the project to the community leaders (Major, Director of health clinic) and asking them to make extensive an invitation to all adult population of the community to participate in the study. Then, we planned visiting days to enroll volunteers in government facilities. We used the Pearson’s chi-squared test and the Fisher exact test (when values were less than 5) for initial comparison of frequencies among groups. Multivariate analysis was used to determine the association between the characteristics of the rural adults and HEV seropositivity. Multivariate analysis modeling included HEV seropositivity as the dependent variable and socio-demographic and behavioral characteristics as independent variables. As criteria for inclusion of independent variables in the multivariate analysis, we considered socio-demographic and behavioral characteristics of participants with a P value equal to or less than 0.20 obtained in the bivariate analysis, and variables with at least 15 positive cases. Odds ratios (OR) and 95% confidence intervals (CI) were calculated by multivariate analysis using backward stepwise logistic regression analysis. A P value less than 0.05 was considered statistically significant. 

### 3.5. Ethical Aspects

The present survey used serum samples and data from a previous study (14), in such previous study the purpose and procedures of the survey were explained to all participants, and a written informed consent was obtained from all of them. This survey was approved by the ethical committees of the Mexican Social Security Institute and the Institute of Security and Social Services of State Workers in Durango City, Mexico.

## 4. Results

One hundred (36.6%) of the 273 rural subjects had anti-HEV IgG antibodies. A selection of socio-demographic and behavioral characteristics of the rural subjects studied and their correlation with HEV seropositivity are shown in Table 1. Two hundred and seventy two subjects were born in Mexico and one in the USA; their mean age was 39.85±17.15 years (range 18-91 years). Of the socio-demographic and behavioral characteristics assessed, the variables gender, age, community of residence, traveled abroad, consumption of untreated water, availability of drinkable water, and form of elimination of excreta had P values < 0.20 by bivariate analysis. Other socio-demographic and behavioral characteristics including educational level, occupation, socio-economic status, type of floorings at home, crowding at home, educational level of the head of the family, raising animals, consumption of any type of meat, consumption of unpasteurized cow milk, unwashed raw vegetables or fruits, eating away from home and contact with soil had P values > 0.20 by bivariate analysis. Two variables including community of residence and pork consumption showed less than 15 positive cases and were not evaluated in the multivariate analysis. Analysis of variables in individual communities showed that HEV seropositivity was associated (P = 0.03) with consumption of untreated water in the community of Villa Montemorelos but not in the other communities. Other socio-demographic and behavioral characteristics of participants including age, educational level, occupation, socio-economic status, crowding at home, raising animals, consumption of unpasteurized cow milk, unwashed raw vegetables or fruits or eating away from home were not associated with HEV seropositivity in individual communities. Further analysis using logistic regression of socio-demographic and behavioral characteristics of rural adults showed that HEV exposure was positively associated with increasing age (OR = 1.04; 95% CI: 1.04-1.05; P < 0.001), consumption of untreated water (OR = 1.92; 95% CI: 1.06-3.46; P = 0.03), and availability of water at home (OR = 1.87; 95% CI: 1.07-3.27; P = 0.02). 

Subjects with blood transfusion history had a higher (20/40: 50%) seroprevalence of HEV exposure than those without such clinical characteristic (80/233: 34.3%) (borderline significance: P = 0.05). Subjects with history of transplantation had a similar seroprevalence (P = 0.62) of HEV infection than those without such clinical characteristic (2/4: 50% vs. 98/269: 36.4%, respectively).

**Table 1.** Bivariate Analysis of Exposure Variables and Seroprevalence of HEV Infection in General Population in Rural Durango ^a^

	Number of subjects tested ^b^	Positive ELISA results	Odds ratio	95% Confidence interval	P Value
**Gender**					
Male	69	30 (43.5)	1.5	0.81-2.67	0.17
Female	204	70 (34.3)	1.0		-
**Age groups, y**					
≤ 30	97	19 (19.6)	1.0		-
31-50	105	41 (39.0)	2.6	1.33-5.23	0.002
> 50	71	40 (56.3)	5.3	2.53-11.19	<0.00001
**Community**					
San Dimas	152	44 (28.9)	1.0		-
Villa Montemorelos	111	50 (45.0)	2.0	1.17-3.47	0.007
Santa Clara	10	6 (60.0)	3.7	0.86-16.49	0.07
**Educational level**					
No education	22	10 (45.5)	1.7	0.19-16.84	0.67
1-6 years	243	87 (35.8)	1.1	0.17-8.96	1
7 or more	6	2 (33.3)	1.0		-
**Occupation**					
Employed	68	29 (42.6)	1.4	0.78-2.59	0.21
Unemployed	204	70 (34.3)	1.0		-
**Socio-economic level**					
Low	188	68 (36.2)	1.0		-
Medium	47	19 (40.4)	1.2	0.59-2.42	0.58
Unknown	38	13 (34.2)	0.9	0.44-1.91	0.81
**Raising farm animals**					
Yes	230	88 (38.3)	1.6	0.72-3.40	0.23
No	42	12 (28.6)	1.0		-
**Traveled abroad**					
Yes	46	25 (54.3)	2.4	1.21-4.82	0.006
No	227	75 (33.0)	1.0		-
**National trips**					
Yes	128	51 (39.8)	1.3	0.77-2.19	0.3
No	145	49 (33.8)	1.0		-
**Pork meat consumption**					
Yes	253	88 (34.8)	0.4	0.13-0.98	0.02
No	20	12 (60)	1.0		-
**Unpasteurized cow milk **					
Yes	181	69 (38.1)	1.2	0.69-2.12	0.47
No	92	31 (33.7)	1.0		-
**Untreated water**					
Yes	83	39 (47)	1.9	1.07-3.29	0.01
No	190	61 (32.1)	1.0		-
**Eating out of home**					
No	51	22 (43.1)	1.0		-
Yes	221	77 (34.8)	0.7	0.36-1.37	0.26
**Soil contact**					
Yes	237	88 (37.1)	1.2	0.53-2.65	0.65
No	36	12 (33.3)	1.0		-
**Availability of drinkable water**					
In home	113	53 (46.9)	2.3	1.33-3.99	0.001
Out of home	148	41 (27.7)	1.0		-
**Excreta disposal**					
Toilet	56	30 (53.6)	2.6	1.35-4.93	0.001
Latrine, open field	204	63 (30.9)	1.0		-
**Crowding**					
No	67	25 (37.3)	1.0		-
Crowded	111	38 (34.2)	0.9	0.44-1.73	0.67
Overcrowded	83	30 (36.1)	1.0	0.46-1.96	0.88
**Education of the head of family**					
Seven or more years	51	15 (29.4)	1.0		-
Up to 6 years	201	75 (37.3)	1.4	0.70-2.94	0.29
**Floor at home**					
Ceramic	24	10 (41.7)	1.6	0.54-4.44	0.36
Concrete	168	61 (36.3)	1.2	0.66-2.32	0.47
Soil	73	23 (31.5)	1.0		-

^a^ Data are presented as No (%).

^b^ Subjects with available data.

## 5. Discussion

In the present study, we found a 36.6% seroprevalence of HEV exposure in adults in rural Durango, Mexico. Such HEV seroprevalence is higher than other HEV seroprevalences in Mexican populations reported so far. There are two previous epidemiological studies on HEV seroprevalence in Mexican populations. In a survey in residents of the central Mexican state of Hidalgo, researchers found a 6.3% seroprevalence of HEV infection (16). While in a national survey in Mexico in subjects from 1 to 29 years old, researchers found a 10.5% seroprevalence of HEV infection, with the highest seroprevalence (14.2%) in people aged 26-29 years old (17). However, comparison of the seroprevalences of HEV infection obtained in the Mexican studies should be interpreted with caution since different assays for diagnosis of HEV exposure were used among the studies. In addition, differences in population characteristics including age and geographic region among the studies account for differences in the seroprevalences obtained. Sampling in previous studies was performed in general population including urban a rural participants. In contrast, in the present study we studied only rural participants. In the national survey, researchers studied a population with a younger age range (1-29 years) than the one (18-91 years) studied in the current survey. Although an association of HEV exposure with low socioeconomic level was found in the previous Mexican studies, we did not find such association in our rural population. Living in rural communities was also a risk factor for HEV exposure in the national survey. There is not data about seroprevalence of HEV exposure in urban Durango, therefore, we cannot compare our results obtained in rural population with others in urban populations in our region. In an international context, comparison of HEV seroprevalences faces the same difficulty because reported epidemiological studies have used various assays for detecting HEV antibodies. Latin America is considered a highly endemic region for hepatitis E (2). In fact, the seroprevalence of HEV infection found in the present study supports such statement. The high (36.6%) seroprevalence of HEV seropositivity found in adults in rural Durango, Mexico suggests a likely higher HEV exposure in rural Mexico than in other Latin American countries. Other Latin American countries including Bolivia (18), Brazil (19, 20), Chile (21), and Venezuela (22) have reported low HEV seroprevalences (1.6%-18.8%) in rural and other populations. The method for sampling in this study was probably the most feasible to enroll participants. Many male adults in rural communities leave their homes to work in the fields during the day time, therefore, a house by house visiting may fail to obtain a representative sample of male participants. However, the sampling method used has the limitation that some ill people might have stayed at home and missed the sampling.

Concerning socio-demographic and behavioral characteristics associated with HEV seropositivity in adults in rural Durango, we found that HEV exposure was positively associated with increasing age, consumption of untreated water and availability of drinkable water at home. The increase of HEV seroprevalence with age found in the present study is consistent with other reports in several populations in Mexico (16, 17) and abroad (23). Of note, HEV seroprevalence did not increase with age in seroepidemiological studies of HEV infection in other Latin American countries including Brazil (19), Chile (21), and Venezuela (22). It is likely that risk factors for HEV infection differ among countries. On the other hand, the association of HEV infection with consumption of untreated water found in the present study agrees with other reports (2, 7, 24) stressing the importance of such transmission route for HEV infection. The seroprevalence of HEV infection varies with employment (25, 26), however, we did not find an association of HEV exposure with occupation. Analysis in individual communities showed that the association of HEV seropositivity with consumption of untreated water occurred in a single community. It is unclear whether other factors contributed for HEV infection in the other two communities. We also found that HEV exposure was associated with availability of drinkable water at home. This finding suggests that HEV exposure occurred at home with contaminated water from the public water supplying systems. In Mexico, water supplied by pipes to houses from public water wells is not suitable for drinking and it should be treated by boiling or other methods before drinking. Some factors evaluated in the present study might compete with each other in explaining the HEV seroprevalence, i.e. having water pipes within the house might be related with a good housing conditions; and treatment of water with good education. However, the criteria used for selection of variables for multivariate analysis reduced the number of variables and allow us to find independent variables associated with HEV exposure. HEV infection has been associated with low socio-economic status (16, 21) and low educational level (17, 27), however, such factors were not associated with HEV infection in the rural subjects studied. Intriguingly, seroprevalence of HEV infection was higher in subjects with history of blood transfusion that those without such clinical characteristic (P = 0.05). Transmission of HEV by blood transfusion may occur (8, 28-30), therefore, further studies to determine the risk for HEV infection by blood transfusion in Mexico are needed. Surveys for blood borne pathogens in rural Durango, Mexico are lacking. Since there is not vaccine against HEV infection in Mexico, results of the present study may help to plan effective preventive measures to avoid HEV exposure in rural populations.

We conclude that the seroprevalence of infection with HEV found in rural populations in Durango is higher than those reported in other Mexican populations. Consumption of untreated water was an important factor for HEV exposure in rural areas in Durango. The correlates of HEV seropositivity found in the present study can be used for an optimal planning of preventive measures against HEV infection. 
